# Changes in the practice of electroconvulsive therapy at Semmelweis University before and during the COVID-19 pandemic

**DOI:** 10.1192/j.eurpsy.2022.1905

**Published:** 2022-09-01

**Authors:** L. Herman, M. Fullajtar, R.I. Zsigmond, J. Réthelyi

**Affiliations:** 1 Semmelweis University, Dept. Of Psychiatry And Psychotherapy, Budapest, Hungary; 2 Semmelweis University, Psychiatry And Psychotherapy, Budapest, Hungary; 3 Semmelweis University, Department Of Psychiatry And Psychotherapy, Budapest, Hungary

**Keywords:** Electroconvulsive therapy, ECT, Covid-19

## Abstract

**Introduction:**

The Department of Psychiatry at Semmelweis University is the largest electroconvulsive therapy (ECT) centre in Hungary, where a total number of around 300 treatments are conducted every year. Certain changes were administered in 2018 and 2019 in our logistics and internal protocols that helped to increase the number of treated patients and improve quality of care. The COVID-19 pandemic caused serious disruptions in the Hungarian mental health care system , therefore there was a realistic fear that many patients who required ECT would not receive this form of tretament.

**Objectives:**

Our goal was to assess the effects of the pandemic on our ECT service, and to analyse whether patients were able to receive treatment, despite the logistical difficulties.

**Methods:**

We retrospectively gathered data from our internal documentation to compare the number of ECT treatments with the previous years. We also had to take into account the fluctuation in our general caseload of psychiatric patients, since our Department acted as a COVID-19 treatment centre for several months.

**Results:**

Total number of ECT treatments decreased in 2020 after a peak in 2019, however the numbers were not much lower compared to the years before changes in 2018. Unfortunately, we see a more direct effect of the 3rd wave of the pandemic.

**Conclusions:**

We can conlude that there is a decrease in the number of ECT treatments due to the pandemic, however, the fact that we still provided service in most parts of 2020 and 2021 for patients with the most severe conditions is a serious achivement.

**Disclosure:**

No significant relationships.

